# Improved pharmacotherapy after revised dosing regimens of two slow-release formulations of benzylpenicillin in an *Actinobacillus pleuropneumoniae* infection model in pigs

**DOI:** 10.1186/s13028-025-00806-9

**Published:** 2025-04-30

**Authors:** Marie Sjölund, Thomas Rosendal, Per Wallgren, Märit Pringle, Ulf Bondesson, Björn Bengtsson, Carl Ekstrand

**Affiliations:** 1 Department of Animal Health and Antimicrobial Strategies, Swedish Veterinary Agency, Ulls Väg 2B, 751 89 Uppsala, Sweden; 2 Department of Clinical Sciences, Faculty of Veterinary Medicine and Animal Science, Swedish University of Agricultural Sciences, Box 7054, 75007 Uppsala, Sweden; 3 Department of Epidemiology, Surveillance and Risk Assessment Epidemiology, Swedish Veterinary Agency, Ulls Väg 2B, 751 89 Uppsala, Sweden; 4 Department of Medicinal Chemistry, Analytical Pharmaceutical Chemistry, Faculty of Pharmacy, Uppsala University, Box 256, 751 05 Uppsala, Sweden; 5 Department of Animal Biosciences, Faculty of Veterinary Medicine and Animal Science, Swedish University of Agricultural Sciences, Box 7023, 75007 Uppsala, Sweden

**Keywords:** Antibiotic, Antimicrobial treatment, Pharmacokinetics, Pleuropneumonia, Porcine, Respiratory disease, Swine, Treatment efficacy

## Abstract

**Background:**

*Actinobacillus pleuropneumoniae* (APP) is a Gram-negative bacterium that causes respiratory disease in pigs, resulting in significant economic losses and reduced animal welfare. In Sweden, the drug of choice for treatment of APP infections is benzylpenicillin. However, limited pharmacokinetic and pharmacodynamic data for benzylpenicillin in pigs have led to variations in recommended dosing regimens. In this study, the impact of different dosing regimens and benzylpenicillin preparations on the progression of APP infection in pigs was investigated. Two experimental trials involving a total of 66 pigs were conducted. Pigs were intranasally inoculated with a pathogenic strain of APP serotype 2, and treatment was initiated upon the appearance of clinical signs. Two intramuscularly administered benzylpenicillin formulations, an aqueous and an oil-based suspension, were used with varying dosing regimens. The clinical outcome was assessed based on respiratory signs and rectal temperature measurements. Blood samples were collected for measuring white blood cell counts, serum antibody levels, and acute-phase protein concentrations. Necropsies were performed to evaluate lung lesions and to reisolate APP.

**Results:**

The results indicated that benzylpenicillin dosing regimens of 20–30 mg/kg administered every 12 h achieved larger benzylpenicillin plasma-exposure compared to the labelled dose of 10–30 mg/kg every 24 h. The oil-based suspension demonstrated superior efficacy compared to the aqueous suspension. Dosing regimens that maintain effective plasma concentrations of benzylpenicillin were shown to have better clinical outcomes as measured by reduced lung lesions at necropsy. Increased benzylpenicillin exposure was associated with a better ranking of overall treatment response.

**Conclusions:**

Several dosing regimens that increased the plasma benzylpenicillin exposure were associated with better clinical success than the labelled doses. The findings support the treatment of APP-infected pigs with optimised benzylpenicillin dosing regimens. Optimising the use of existing antibiotics is crucial given the limited development of new antimicrobial agents and the need to combat antimicrobial resistance with regards to both human and animal health.

## Background

*Actinobacillus pleuropneumoniae* (APP) is a Gram-negative bacterium that can cause respiratory infections in pigs leading to reduced animal welfare and financial losses [1–4]. During peracute outbreaks of actinobacillosis, mortality might be high without previous clinical signs of disease. Acutely diseased pigs develop fever, cough, anorexia, and listlessness. In contrast, pleuritis detected at slaughter may be the only evidence of infection in pigs affected by chronic actinobacillosis [5]. Pigs with acute APP infection require treatment and since APP is a common porcine pathogen, pigs are regularly treated with antimicrobials due to actinobacillosis. However, antimicrobial resistance, which is a threat to both animal and human health, is correlated with the use of antibiotics [6–8].

Within the European Union, regulation (EU2019/6) allows for critically important antibiotics to be reserved for humans. The Antimicrobial Advice Ad Hoc Expert Group (AMEG) by the European Medicines Agency has set up advice for the use of antibiotics in veterinary medicine to manage potential risks to humans. Simultaneously, incentives for developing new classes of antibiotics are weak [9]. Therefore, it is essential that the efficacy of already-marketed antibiotics used in veterinary medicine is maintained [10–12]. Benzylpenicillin is a narrow-spectrum antibiotic discovered in the late 1920 s and introduced for use in animals in the 1950 s. Benzylpenicillin has been categorized as category-D (prudent use) by the AMEG. Due to restrictive use and measures to counteract the spread of resistant bacteria, benzylpenicillin is still the drug of choice for treating the most common infections in livestock production in Sweden [12]. Consequently, injectable benzylpenicillin dominates the overall use of antibiotics for pigs in Sweden [13, 14].

Pharmacokinetic and pharmacodynamic (PK/PD) data on benzylpenicillin in pigs are sparse, which may be the reason for variation in recommended dosing in the literature [15–17]. In Sweden, the labelled dose of 20 mg/kg once daily for two of the procaine benzylpenicillin products on the market, Ethacilin vet (Intervet AB, Stockholm, Sweden) and Penovet vet (Boehringer Ingelheim Vetmedica, Malmö, Sweden), could not be shown to be effective treatments for APP in experimental studies [18–20]. The lack of clinical efficacy was not related to antimicrobial resistance since isolates of APP from Swedish pigs, including the challenge strains, are susceptible to benzylpenicillin in vitro [14]. The poor response in the aforementioned experimental infections could be due to insufficient exposure as clinical experience indicates that higher doses are effective in the field.

Beta-lactam antibiotics, such as benzylpenicillin, are classified as time-dependent antibiotics [21]. For this antibiotic class, a significant increase in concentration above the minimum inhibitory concentration (MIC) has a minimal effect on the rate and extent of bacterial killing [22]. Instead, the time that the free plasma concentration exceeds the MIC over the dosing interval (fT > MIC) is the best predictor of the antimicrobial effect [23]. For susceptible Gram-negative bacteria, fT > MIC values over 40% of the dosing interval are considered bacteriostatic, and up to 70% might be necessary for maximal bactericidal effects [24]. For benzylpenicillin, a clinical point estimate for bactericidal effects is fT > MIC of 50% of the dosing interval [21]. This might not be achieved with the labelled dose since the MIC of benzylpenicillin for susceptible APP isolates is high (0.25–0.50 mg/L) [14].

The labelled dosing regimens (10–30 mg/kg once daily) is assumed to maintain a free plasma concentration above MIC for at least 12 h. In a recent exposure study in pigs, this was not achieved with the labelled dose of aqueous procaine benzylpenicillin suspension [25]. However, the dose of 30 mg/kg for a marketed suspension of procaine benzylpenicillin in oil (Ultrapen, N-vet AB, Uppsala, Sweden) was sufficient. The results from that study also indicated that effective plasma exposure was achieved with the dosing regimen 20–30 mg/kg every 12 h of the aqueous suspensions of procaine benzylpenicillin. Despite those results supporting anecdotal field reports, no scientific evidence supports successful treatment of APP-infected pigs with benzylpenicillin.

The objective of this study was to investigate the impact of different dosing regimens and different procaine benzylpenicillin preparations on the disease progression in pigs experimentally infected with APP.

## Methods

### Animals and housing facilities

A total of 66 pigs originating from one specific pathogen-free (SPF) herd were used in the study [25]. The pigs were either purebred Yorkshire or two- or three-breed hybrids of Yorkshire, Landrace, or Duroc. All pigs were 11 to 13 weeks old and weighed between 33 and 48 kg at the beginning of the two trials.

The study involved two different trials previously described that were carried out in the experimental facilities of the Swedish Veterinary Agency (SVA): trial 1 and trial 2 [25]. Briefly, in trial 1, 30 pigs from six litters were allocated to one of five treatment groups with six pigs in each group. In trial 2, 36 pigs were allocated to six treatment groups of six pigs. The groups were adjusted to include an even distribution of sex, weight on arrival, and litter of origin. Plasma benzylpenicillin concentration data from the treated pigs were included in a previous pharmacokinetic study [25]. The groups were housed in separate rooms with separate ventilation and manure systems as previously described [26]. The use of live animals in these experiments was done in accordance with Swedish legislation and was approved by the regional ethical committee in Uppsala (License C 37/16) and by the Swedish Medical Products Agency (License no. 5.1–2016–16737).

### Feeding regimen, body weights and daily weight gain

The pigs had free access to water through a nipple drinker and, were fed a dry commercial feed for finishing pigs (trial 1: Sluka slaktgrisfoder, trial 2: Slaktfor, Lantmännen, Malmö, Sweden) ad libitum by feeders (Piggomat, Skälby Maskin, Enköping, Sweden). The crushed pelleted feed, mainly constituting barley and wheat, had a crude protein level of 12.6% (Sluka, trial 1) or 13% (Slaktfor, trial 2) with 8.1 g lysine (both trials), and 2.4 (Sluka, trial 1) or 2.5 g (Slaktfor, trial 2) methionine per kg. The net energy was 9.3 MJ per kg feed for both trials. The feed intake was defined at the group level as the average feed consumed per pig.

All pigs were weighed (Danvægt Model 203E, Danvægt, Hinnerup, Denmark) individually on the day of arrival (day −7), and on days: 1, 6, 13, and 16 post infection (pi). The average daily weight gain (ADWG) was calculated for each pig.

### General experimental design

After an acclimatization period of one week, all pigs in the trial groups were inoculated intranasally with 10^11^ colony-forming units (CFU) of an APP serotype 2 (strain 700/89, SVA) grown overnight on Bacto pleuropneumoniae-like organism (PPLO) agar at 37 °C in a humid atmosphere with 5% CO_2_ and harvested into 0.9% saline. The bacterial suspension was inoculated into the nasal cavity of restrained but non-sedated pigs using a 2 ml syringe. The nostril was blocked following the inoculation for five consecutive inhalations. The MIC of benzylpenicillin for the APP strain used was 0.5 mg/L, (VetMIC, SVA, Swedish Veterinary Agency, Sweden).

Following inoculation (day 0), all pigs were monitored including observation for the appearance of respiratory signs. Treatments were initiated in all pigs when clinical signs of an APP infection such as fever, coughing or increased respiratory rate, or loss of appetite were observed in the majority of pigs with an average clinical score of 1. Benzylpenicillin treatment was initiated at 17.5 and 20 h in trial 1 and 2, respectively. To evaluate the clinical outcome in relation to different dosing regimens, two different benzylpenicillin formulations were used for treatment. The two formulations used were: Ethacilin vet (ETH) (Intervet AB, Sollentuna, Sweden) with a concentration of 300 mg/mL procaine benzylpenicillin in an aqueous suspension, and Ultrapen vet (UPA) (N-vet, Uppsala, Sweden), with a concentration of 300 mg/mL procaine benzylpenicillin in an oil-based suspension. Dosing regimens for both trials of the study are shown in Table 1. All treatments were given as intramuscular (IM) injections in the lateral neck according to previous publication [25].

**Table 1:** Dosing regimens for two benzylpenicillin formulations used to evaluate treatment efficacy

Trial	Group name	Medicinal product	Loading dose (mg/kg)	Maintenance dose (mg/kg)	Dosing interval	Treatment duration (days)
1	ETH20-2 × 3	Ethacilin vet	–	20	Every 12 h	3
1	ETH20-1 × 3	Ethacilin vet	–	20	Every 24 h	3
1	ETH30-1 × 3	Ethacilin vet	–	30	Every 24 h	3
1	UPA30-1 × 3	Ultrapen vet	–	30	Every 24 h	3
2	ETH30-2 × 5	Ethacilin vet	–	30	Every 12 h	5
2	UPA30-1 × 5	Ultrapen vet	–	30	Every 24 h	5
2	UPA30-1 × 3	Ultrapen vet	–	30	Every 24 h	3
2	UPA60 + UPA30-1 × 2	Ultrapen vet	60	30	Every 24 h	3
2	ETH30 + UPA30-1 × 3	Ethacilin vet/ Ultrapen vet	30^1^	30	Every 24 h	3.5

^1^Ethacilin vet 30 mg/kg was administered twelve hours before the first Ultrapen vet administration

Pigs were before treatment inoculated intranasally with an *Actinobacillus pleuropneumoniae* (APP) serotype 2 strain. The two formulations used were: Ethacilin vet (ETH) (Intervet AB, Sollentuna, Sweden) containing 300 mg/ml procaine benzylpenicillin in an aqueous suspension, and Ultrapen vet (UPA) (N-vet, Uppsala, Sweden), containing 300 mg/ml procaine benzylpenicillin in an oily suspension. The treatment group nomenclature is: the medicinal product; dose in mg/kg; dose frequency (once or twice daily) and treatment duration in days. For example, ETH20-2 × 3 should read as: procaine benzylpenicillin in a water suspension 20 mg/kg bodyweight twice daily for three days

### Clinical scoring and measurement of rectal temperatures

A previously established scoring system for respiratory signs, from 0 to 3, was used to assess the clinical outcome of the infection [18, 19]. In brief, 0 denoted absence of respiratory signs; 1 mild clinical signs (moderately forced breathing); 2 moderate clinical signs (moderately forced breathing combined with sporadic cough or severely forced breathing without cough); and 3 severe clinical signs (severely forced breathing and coughing). Clinical signs were scored by a single observer to avoid inter-observer error.

The rectal temperature of the pigs was measured using a high-speed digital thermometer (Braun Age Precision™, Kaz Europe Sàrl, Lausanne, Switzerland). After the inoculation, rectal temperature was recorded at least twice daily until day four of the trial. Thereafter, the temperature was recorded once daily until day 10 and again on day 13.

### Sampling procedures and sample analyses

Pigs were restrained by the snout to allow for sampling. Blood samples were collected from the jugular vein in 7 ml evacuated plastic tubes (BD Vacutainer®, Stockholm, Sweden) containing an ethylenediaminetetraacetic acid (EDTA) additive as well as tubes with no additive. Samples without additive were centrifuged for 10 min at 800 g, after which the serum was transferred to plastic storage tubes (2 ml Screw Cap Micro Tubes, Sarstedt AB, Helsingborg, Sweden). Samples were then stored at –20 °C until analysis. Blood samples in trial 1 were collected on the following days: −1, 1, 2, 3, 4, 5, 6, 10, 13 (group ETH20-2 × 3 was not sampled on day 13) and day 16. Blood samples in trial 2 were collected on the following days: −1, 1, 2, 3, 4, 5, 6, 11, and 16. For use in the previously published pharmacokinetic study using the same experiment [25], additional blood was drawn also at several occasions the first 24 h after benzylpenicillin administration. Those samples were not used in the present study.

An indirect ELISA was used to measure serum antibodies to APP serotype 2, with a cut-off value for a positive reaction in sera diluted 1/1000 defined as A450 nm = 0.50 [27]. Serum amyloid A (SAA) was analysed with a commercial kit according to the manufacturer’s instructions (Phase SAA, Tridelta Development Ltd, Maynooth, Ireland).

Samples with EDTA additive were used for total white blood cell counts (WBC) using an automatic analyser (Exigo, Boule, Stockholm, Sweden) on the day of sampling.

### Necropsies and re-isolation of A. pleuropneumoniae serotype 2

All pigs were euthanized and necropsied on day 16. The presence of pericarditis was recorded. The extent of pneumonia and pleurisy was defined as the percentage of the total affected lung volume and lung surface area, respectively, using a previously described protocol [18]. Samples for bacterial cultivation were collected from affected lung tissue, lung abscesses (if present), bronchial lymph nodes, and tonsils from each pig using sterile 10 ml inoculating loops. The loops were inserted into incisions of the affected tissue and scraped against the tissue to obtain enough material. The samples were then immediately streaked on blood agar plates, cross-inoculated with a single streak of a nurse strain of *Staphylococcus aureus* at 37 °C in a humid atmosphere with 5% CO_2_, and read after 24 and 48 h. Susceptibility testing for benzylpenicillin was performed on re-isolated APP bacteria (VetMIC, SVA, Swedish Veterinary Agency, Sweden).

### Statistical analysis

Data on treatments of individual animals and the measured observations over the study period including clinical score, rectal temperature, WBC, serum APP antibody concentration, acute phase protein concentration, body weight, sex, and litter identifier were summarized. The measurements from each pig were distributed over as many as 46 time-points from seven days prior to experimental infection to 16 days post-infection. Observations of pulmonary lesions at necropsy including pneumonia, pleurisy, and pericarditis were also summarized. Each variable was visualized as a time series of parallel box plots stratified by treatment and trial.

The associations between each of the clinical and laboratory outcomes and different treatments were tested, after adjusting for repeated measures of individual pigs, using the generalized estimating equation (GEE) [28]. The GEE method is well suited for analysis of time series data and accounts for correlation between repeated measures over time. Associations between the outcomes measured at necropsy and different treatments were tested using a generalized linear mixed model (GLMM) [29] adjusted for repeated measurement within the same litter and the experimental effect. All statistics were done in the R statistical software version 4.3.2 (R Core team 2023). GLMM was used in this case since necropsy data was collected at only one time point and the trial and litter effects could be suitably captured in a random intercept term. Each model included the two separate untreated groups as a reference and each treatment was ranked by the magnitude of its apparent beneficial association with improvement in pig health as measured by each outcome. Statistically significance differences were considered to be those with P ≤ 0.05. To identify the potentially most effective treatment, a treatment with a favourable ranking in several parameters (overall treatment-response ranking) was considered superior to one with a lower ranking.

Plasma benzylpenicillin concentrations used in this study were previously published [25] and included in the current evaluation of treatment efficacy. Measurement intervals for each pig where the plasma concentration was decreasing were used to calculate a decay parameter assuming exponential decay of the plasma concentration of benzylpenicillin. The median calculated decay was used to interpolate the plasma concentration for each pig between measurements during the study period. The required therapeutic concentration was considered to be a total plasma concentration exceeding 1 mg/L because the fraction protein bound is approximately 45% [30]. An effective treatment was considered one where the plasma concentration did not fall below this threshold for greater than 50% of the dose interval. For each pig, the ‘effective treatment time’ was calculated as the longest period, in hours, above 1 mg/L benzylpenicillin plasma concentration that was uninterrupted by a dip below this level for greater than 50% of the dosing interval. The association between effective treatment time and the following variables: fever at 48 h post-challenge; the percent of the lung affected by pneumonia; pleuritis and thoracic adhesions was tested by a generalized linear model with a random effect to control for clustering within litter and experiment.

## Results

Three pigs became lame before inoculation. One pig in group ETH30 + UPA30-1 × 3 became lame with increased rectal temperature (41.2 ⁰C) at day −3. Flunixin meglumine (5 mg/kg) was administered intramuscularly at days −3, −2 and −1 to that pig. One pig in group UPA30-1 × 3 became lame with increased rectal temperature (40.6 ⁰C) at day −3. Flunixin meglumine (5 mg/kg) was administered intramuscularly at days −3, −2, −1 and 0 to that pig. One pig in the untreated group became lame at day −2. Flunixin meglumine (5 mg/kg) was administered intramuscularly at days −2 and −1 to that pig. Rectal temperature was normal at day 0 in these three pigs. All other drug administrations were consistent with the study protocol.

### Respiratory clinical scores

**Fig. 1:**
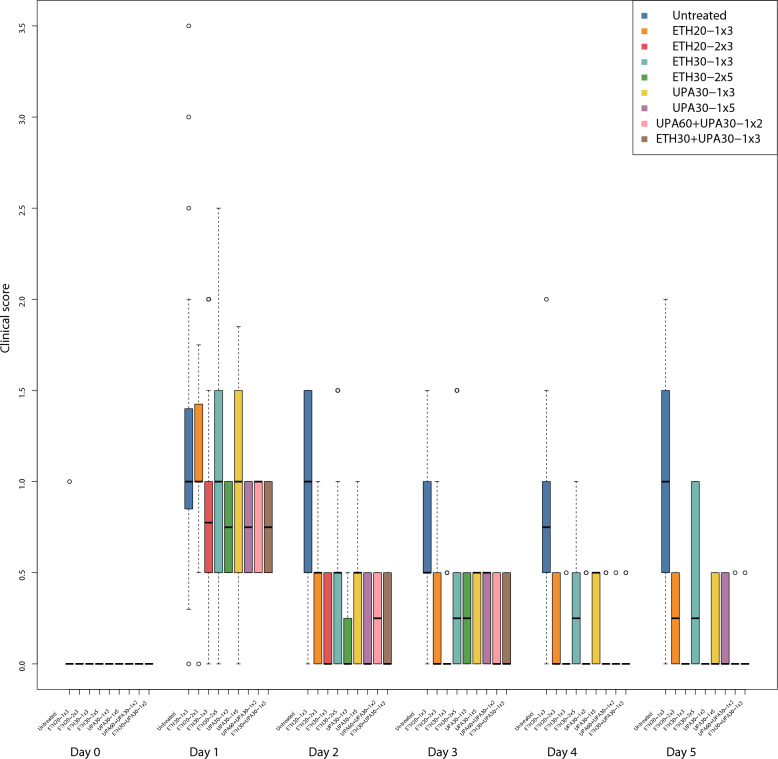
Parallel Tukey boxplots of the clinical score in pigs. The boxplot is over the first 5 days after inoculation (Day 0) with *Actinobacillus pleuropneumonia* serotype 2. Benzylpenicillin treatment was initiated at the onset of clinical signs which occurred within 24 h after inoculation. The treatment group nomenclature is: the medicinal product, either procaine benzylpenicillin in a water suspension (ETH) or procaine benzylpenicillin in an oil suspension (UPA); dose in mg/kg; dose frequency (once or twice daily) and treatment duration in days. For example, ETH20-2 × 3 should read as: procaine benzylpenicillin in a water suspension 20 mg/kg bodyweight twice daily for three days

No clinical signs of respiratory disease were observed in the pigs during the acclimatization period prior to the inoculation. Pigs with clinical signs were found in all groups following the inoculations with APP serotype 2 (Fig. 1). All pigs, except one pig in trial 1, showed clinical signs of respiratory disease within 24 h following inoculation. Disease severity and progression varied between individuals with one pig in trial 1 showing per acute disease progression. The first clinical signs in this pig were observed three hours after inoculation. The clinical score for this pig was at that time 1. This pig died peracutely 19 h after inoculation between times of observations. This pig was not included in the data analysis. The remaining pigs were euthanized at the end of the experiment for post-mortem examination, consistent with the study protocol.

All treatments were associated with a significantly lower clinical score than the untreated groups and the between-trial effect was not significant. None of the dosing regimens were significantly different from one another but the regimen with the best ranking was ETH30-2 × 5 with a clinical score of 0.62 units (CI: 0.5, 0.78) lower than the untreated groups (Table 2). ETH30 + UPA30-1 × 3 and UPA60 + UPA30-1 × 2 were 2nd and equally ranked.

**Table 2:** The treatment-response ranking of the response to each treatment by its effect on the outcome

Treatment	Clinical score	Antibody response	Body temperature	Serum amyloid A	WBC	Pneumonia	Pleuritis	Thoracic adhesions	Mean
ETH20-1 × 3	7	6	8	–	–	–	–	–	7.6
ETH20-2 × 3	5	-	4	–	4	6	4	4	5.4
ETH30-1 × 3	8	5	2	–	–	7	7	–	6.6
ETH30-2 × 5	1	3	4	1	2	3	2	5	2.6
UPA30-1 × 3	6	-	3	–	3	5	6	3	5.3
UPA30-1 × 5	4	1	6	–	1	4	5	6	4.4
UPA60 + UPA30-1 × 2	2	4	1	2	6	1	3	1	2.5
ETH30 + UPA30-1 × 3	2	2	6	–	5	2	1	2	3.4

The numbers in the table indicate the rank of the treatment effect as measured by the generalized estimating equation (GEE) with adjustment for the experiment effect and repeated measures within pig. A hyphen indicates that the treatment effect was not significantly different than the untreated group and therefore was not ranked. The treatment group nomenclature is: the medicinal product, either procaine benzylpenicillin in a water suspension (ETH) or procaine benzylpenicillin in an oil suspension (UPA); dose in mg/kg (20, 30 or 60 mg/kg); dose frequency (once or twice daily) and treatment duration in days (3 or 5 days). For example, ETH20-2 × 3 should read as: procaine benzylpenicillin in a water suspension 20 mg/kg bodyweight twice daily for three days. The last column is the mean ranking for each treatment group which gives an indication of the total ranking of the treatment across clinical outcomes. The mean was calculated by assigning a value of 8 to each non-significant (–) response indicating that it was equivalent to being ranked lowest

### Rectal temperatures

**Fig. 2:**
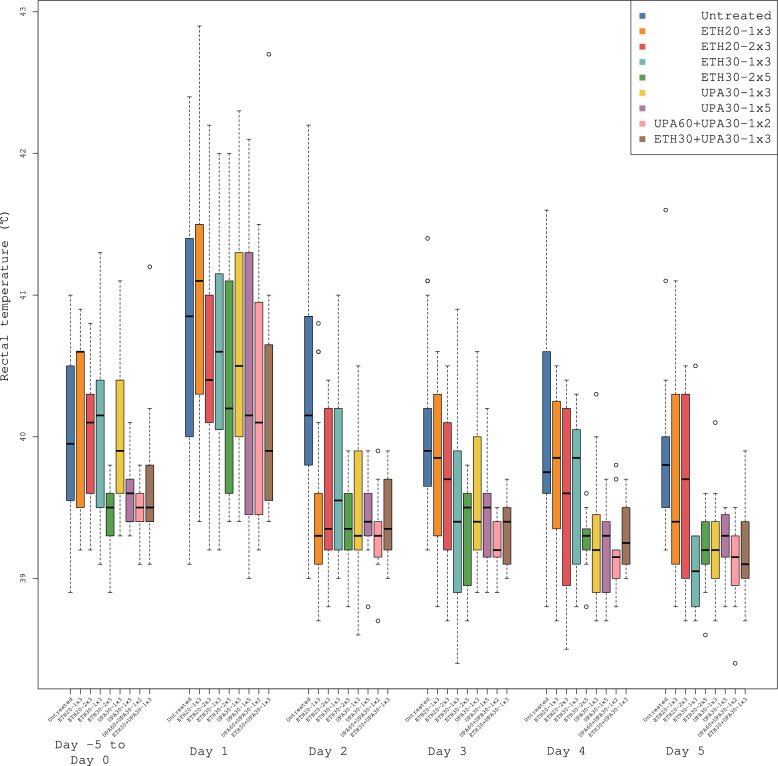
Parallel Tukey boxplots of the rectal temperature in pigs. The boxplot covers the acclimatization period (Day –5 to Day 0) and the first five days after inoculation (Day 0) with *Actinobacillus pleuropneumonia* serotype 2. Temperatures included in ‘Day 1’ range from ~ 6 h post-inoculation to 24 h post-inoculation; subsequent measurements were taken at ~ 24-h intervals. Benzylpenicillin treatment was initiated at the onset of clinical signs which occurred within 24 h after inoculation. The treatment group nomenclature is: the medicinal product, either procaine benzylpenicillin in a water suspension (ETH) or procaine benzylpenicillin in an oil suspension (UPA); dose in mg/kg; dose frequency (once or twice daily) and treatment duration in days. For example, ETH20-2 × 3 should read as: procaine benzylpenicillin in a water suspension 20 mg/kg bodyweight twice daily for three days

Rectal temperatures were ≤ 40 °C prior to inoculation for all groups in both trials. Inoculation increased rectal temperature in all groups (Fig. 2). All dosing regimens were associated with significantly lower rectal temperatures than the untreated groups. The rectal temperatures in trial 2 were 0.48 °C (CI: 0.32 °C, 0.65 °C) lower than in trial 1. The between-trial effect was not significant. None of the dosing regimens were significantly different from one another but the regimen with the best ranking was UPA60 + UPA30-1 × 2 which had a 0.43 °C (CI: 0.28, 0.57) lower rectal temperature than the untreated groups. ETH30-1 × 3 and UPA30-1 × 3, ranked 2nd and 3rd (Table 2).

### Serum Amyloid A

**Fig. 3:**
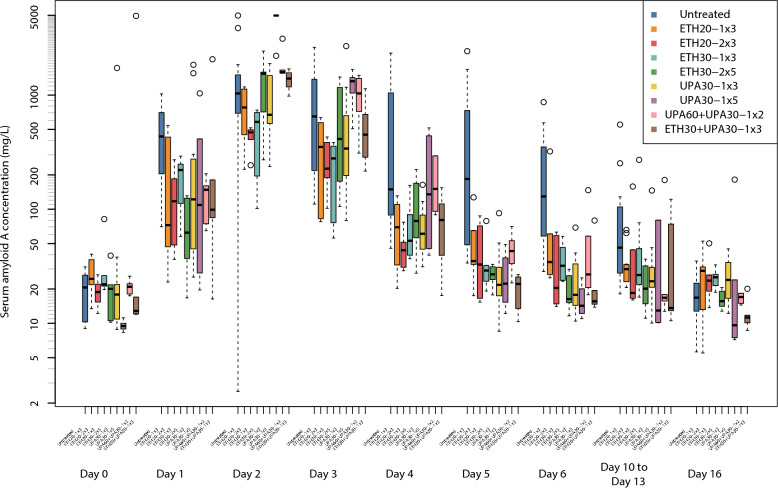
Parallel Tukey boxplots of serum amyloid A blood concentration in pigs. The serum amyloid concentrations were measured after inoculation (Day 0) with *Actinobacillus pleuropneumonia* serotype 2. Benzylpenicillin treatment was initiated at the onset of clinical signs which occurred within 24 h after inoculation. The treatment group nomenclature is: the medicinal product, either procaine benzylpenicillin in a water suspension (ETH) or procaine benzylpenicillin in an oil suspension (UPA); dose in mg/kg; dose frequency (once or twice daily) and treatment duration in days. For example, ETH20-2 × 3 should read as: procaine benzylpenicillin in a water suspension 20 mg/kg bodyweight twice daily for three days

The SAA concentrations increased post inoculation in all groups (Fig. 3). The ETH30-2 × 5 and UPA60 + UPA30-1 × 2 dosing regimens were associated with significantly lower SAA concentrations than the untreated groups. The ETH30-2 × 5 regimen was the best ranked and the SAA concentration was 451.9 mg/L (CI: 159.5 mg/L, 744.3 mg/L) lower than the untreated groups (Table 2). The SAA concentrations in trial 2 were 471 mg/L (CI: 262 mg/L, 680 mg/L) higher than trial 1. None of the dosing regimens were significantly different from one another.

### Total white blood cell counts

**Fig. 4:**
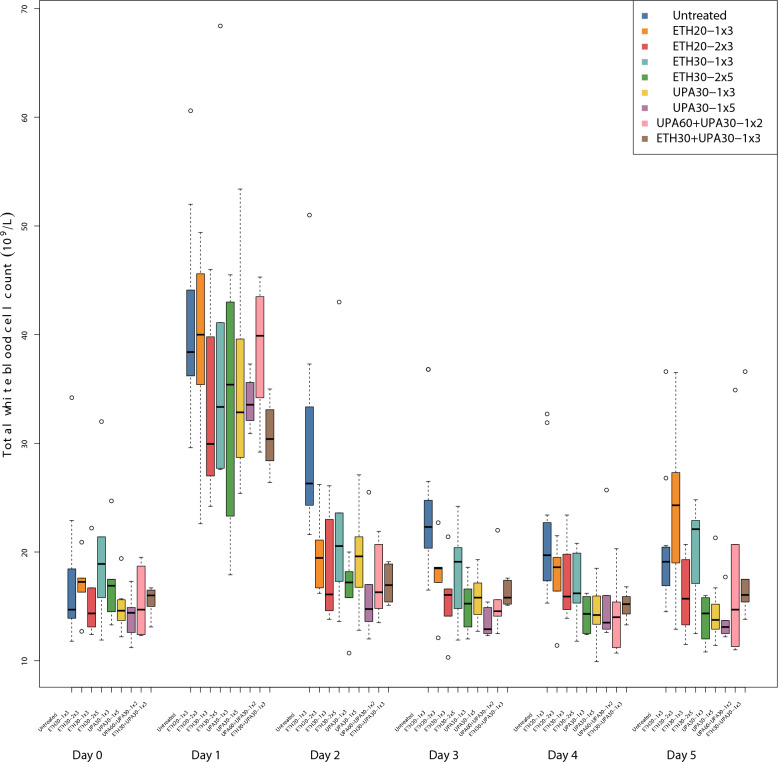
Parallel Tukey boxplots of the total white blood cell count in pigs. The boxplots are made of data from the first 5 days after inoculation with *Actinobacillus pleuropneumonia* serotype 2. Benzylpenicillin treatment was initiated at the onset of clinical signs which occurred within 24 h after inoculation (Day 0). The treatment group nomenclature is: the medicinal product, either procaine benzylpenicillin in a water suspension (ETH) or procaine benzylpenicillin in an oil suspension (UPA); dose in mg/kg; dose frequency (once or twice daily) and treatment duration in days. For example, ETH20-2 × 3 should read as: procaine benzylpenicillin in a water suspension 20 mg/kg bodyweight twice daily for three days

Total WBC increased in all groups after inoculation (Fig. 4). All dosing regimens except ETH20-1 × 3 and ETH30-1 × 3 were associated with a significantly lower WBC than the untreated groups and the between-trial effect was not significant. None of the dosing regimens were significantly different from one another but, the regimen with the best ranking was UPA30-1 × 5 with a 6.2 × 10^9^/L (CI: −8.47, −3.9) lower WBC than the untreated groups. ETH30-2 × 5 and UPA30-1 × 3 were ranked 2nd and 3rd (Table 2).

### Serum antibodies to A. pleuropneumoniae serotype 2

**Fig. 5:**
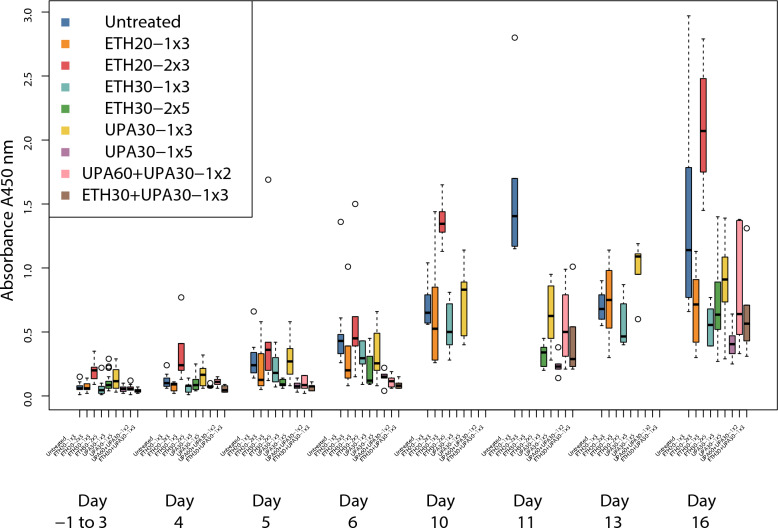
Parallel Tukey boxplots of serum antibodies to *Actinobacillus pleuropneumonia* serotype 2 (APP2) in pigs. Serum antibodies were measured after inoculation (Day 0) with APP2. Results are aggregated from one day before inoculation to day 3 for readability since the results are similar for this range. Benzylpenicillin treatment was initiated at the onset of clinical signs which occurred within 24 h after inoculation. The treatment group nomenclature is: the medicinal product, either procaine benzylpenicillin in a water suspension (ETH) or procaine benzylpenicillin in an oil suspension (UPA); dose in mg/kg; dose frequency (once or twice daily) and treatment duration in days. For example, ETH20-2 × 3 should read as: procaine benzylpenicillin in a water suspension 20 mg/kg bodyweight twice daily for three days

The reduction of absorbance is in absolute terms of absorbance, not a proportion. All dosing regimens except ETH20-2 × 3 and UPA30-1 × 3 were associated with significantly lower serum antibodies than the untreated groups (Fig. 5). The between-trial effect was not significant. None of these dosing regimens were significantly different from one another but, the regimen with the best ranking (lowest serum antibodies) was UPA30-1 × 5, which had 0.38 lower absorbance than the untreated groups (CI: 0.21, 0.54). Treatment groups ETH30 + UPA30-1 × 3 and ETH30-2 × 5 were ranked 2nd and 3rd (Table 2).

### Feed intake

**Fig. 6:**
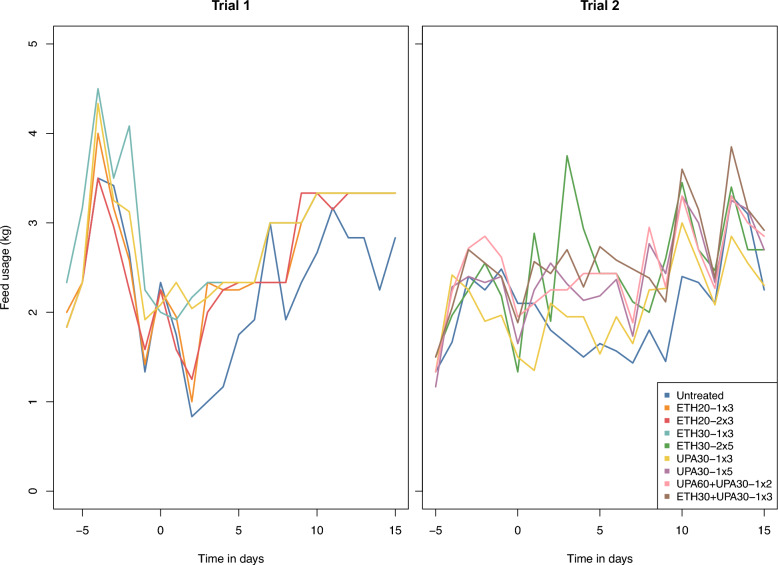
Mean feed consumption per pig per day before and after inoculation with *Actinobacillus pleuropneumonia* serotype 2. Benzylpenicillin treatment was initiated at the onset of clinical signs which occurred within 24 h after inoculation (Day 0). The treatment group nomenclature is: the medicinal product, either procaine benzylpenicillin in a water suspension (ETH) or procaine benzylpenicillin in an oil suspension (UPA); dose in mg/kg; dose frequency (once or twice daily) and treatment duration in days. For example, ETH20-2 × 3 should read as: procaine benzylpenicillin in a water suspension 20 mg/kg bodyweight twice daily for three days

Statistical analysis was not done on feed intake because the data was recorded at the group level. Feed intake numerically decreased in the days after inoculation for all treatment groups except ETH30-1 × 3 and UPA30-1 × 3 (Fig. 6). In general, feed intake returned to pre-inoculation levels in all groups within the observation period with a tendency for a later return for the untreated groups.

### Average daily weight gain

**Fig. 7:**
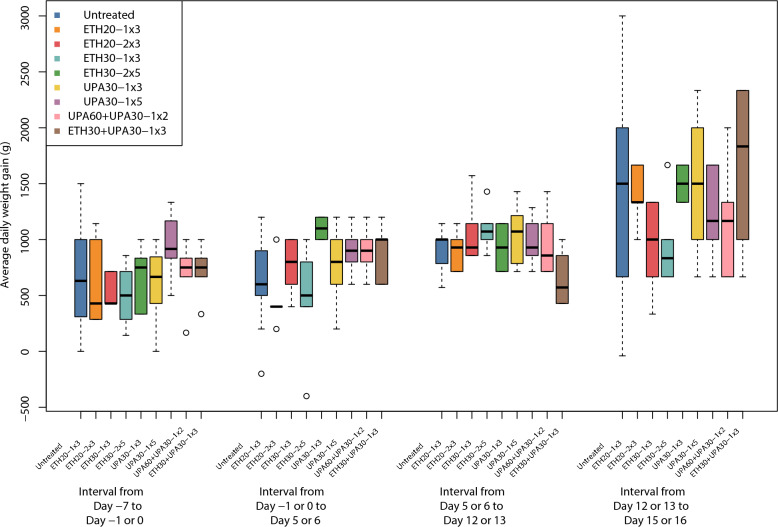
Parallel Tukey boxplots of average daily weight gain in pigs. Data was collected after inoculation (Day 0) with *Actinobacillus pleuropneumonia* serotype 2. Benzylpenicillin treatment was initiated at the onset of clinical signs which occurred within 24 h after inoculation. The treatment group nomenclature is: the medicinal product, either procaine benzylpenicillin in a water suspension (ETH) or procaine benzylpenicillin in an oil suspension (UPA); dose in mg/kg; dose frequency (once or twice daily) and treatment duration in days. For example, ETH20-2 × 3 should read as: procaine benzylpenicillin in a water suspension 20 mg/kg bodyweight twice daily for three days

None of the dosing regimens resulted in significantly different ADWG than the untreated groups and the between-trial effect was not significant. There was, however, a tendency for higher ADWGs in the treated groups (Fig. 7). None of the dosing regimens were significantly different from one another but, the regimen with the highest ranking (largest ADWG) was ETH30-1 × 3.

### Post-mortem examinations

The extent of macroscopic lesions of pneumonia, pleuritis, and thoracic adhesions, measured as the percent of the respective surface areas, are presented in Table 3. The reduction of lesions compared to the untreated group is expressed as a percent in absolute units. This is the additive effect on the percent scale, i.e. a reduction of 20% would mean that the untreated group had lung lesions affecting 40% of the lung and the treated group 20%. The extent of all observed lesions was significantly lower in all benzylpenicillin-treated groups except thoracic adhesion in groups: ETH30-1 × 3 and UPA30-1 × 5, compared to untreated pigs. None of the dosing regimens were significantly different from one another. For pneumonia lesions, the dosing regimen with the highest rank (lowest percent pneumonia) was UPA60-UPA30-1 × 2 (−40.8%; CI: −57.7%, −23.9%) followed by ETH30-UPA30-1 × 3 and ETH30-1 × 5. Similarly, for pleuritis at necropsy, the dosing regimen with the highest rank (lowest percent pleuritis) was ETH30-UPA30-1 × 3 (−42.5%; CI: −58.6%, −26.4%) followed by ETH30-2 × 5 and UPA60 + UPA30-1 × 2. Finally, for thoracic adhesions, the dosing regimen with the highest rank (lowest thoracic adhesions) was UPA60 + UPA30-1 × 2 (−20.8%; CI: −34.6%, −7.1%) followed by ETH30 + UPA30-1 × 3 and UPA30-1 × 3 (Table 2).

**Table 3:** The extent of median macroscopic lesions of pneumonia, pleuritis, and thoracic adhesions

Trial	Treatment group	Extent of pneumonia (%)	Extent of pleuritis (%)	Thoracic adhesions (%)
1	ETH20-2 × 3	13 (1–27)	5 (0–16)	2 (0–15)
1	ETH20-1 × 3	12 (3–32)	9 (0–30)	0 (0–15)
1	ETH30-1 × 3	16 (3–62)	16 (3–62)	20 (0–55)
1	UPA30-1 × 3	25 (9–88)	19 (5—71)	0 (0–50)
1	Untreated	44 (39–99)	50 (30–100)	10 (3–40)
2	ETH30-2 × 5	2 (0–15)	0 (0–9)	0 (0–30)
2	UPA30-1 × 5	0 (0–29)	0 (0–28)	0 (0–45)
2	UPA30-1 × 3	1 (0–13)	1 (0–7)	0 (0–10)
2	UPA60 + UPA30-1 × 2	0 (0–2)	1 (0–5)	0 (0–2)
2	ETH30 + UPA30-1 × 3^1^	1 (0–4)	0 (0–0)	0 (0–3)
2	Untreated	29 (4–40)	29 (8–45)	18 (3–65)

^1^ Ethacilin vet 30 mg/kg was administered twelve hours before the first Ultrapen vet administration

Pigs were challenged with an intranasal inoculation of *Actinobacillus pleuropneumoniae* serotype 2 and then treated with different dosing regimens of procaine benzylpenicillin intramuscularly. The treatment group nomenclature is: the medicinal product, either procaine benzylpenicillin in a water suspension (ETH) or procaine benzylpenicillin in an oil suspension (UPA); dose in mg/kg; dose frequency (once or twice daily) and treatment duration in days. For example, ETH20-2 × 3 should read as: procaine benzylpenicillin in a water suspension 20 mg/kg bodyweight twice daily for three days

### Bacterial culture at post-mortem examination

*Actinobacillus pleuropneumoniae* was cultured from affected lung tissue from a total of five pigs. Susceptibility testing was performed in one re-isolated strain, which was susceptible to penicillin. Two culture-positive pigs were found in groups ETH20-2X3, and the untreated group in trial 1. One pig was culture-positive in the UPA30-1X3 group. Culture-positive lung abscesses were found in 12 pigs in total, one pig in treatment group ETH20-2X3, two pigs in treatment groups ETH30-1 × 3, ETH30 + UPA30-1 × 3, UPA30-1 × 5 and UPA30-1 × 3 respectively, three pigs in the treatment group UPA60 + UPA30-1 × 2, and four pigs in treatment groups ETH30-2 × 5 and untreated pigs respectively. Cultures from lymph nodes were all negative. In addition, APP was cultured from the lung, bronchial lymph node, and pericardium from the pig that died peracutely.

### Effective treatment time

**Fig. 8:**
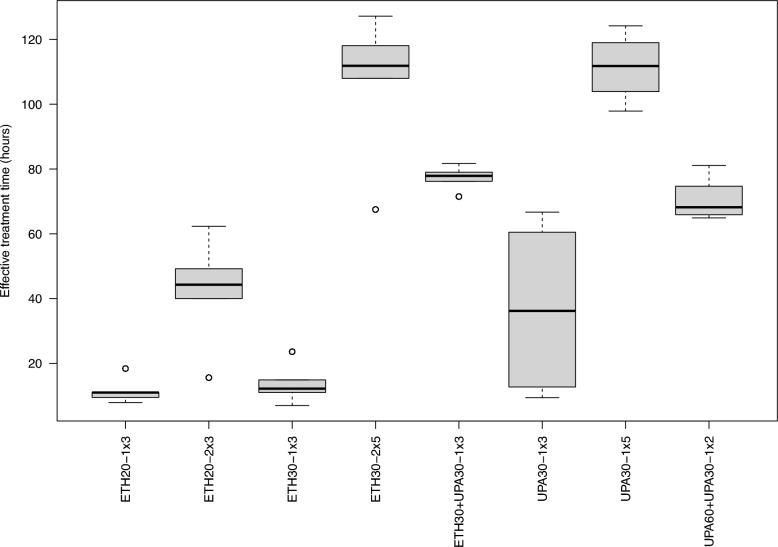
The distribution of effective treatment time in hours for each of the treatment regimens. Represents the number of hours where total benzylpenicillin plasma concentration exceeded 1 mg/L without an interruption of greater than 50% of the dosing interval. The treatment group nomenclature is: the medicinal product, either procaine benzylpenicillin in a water suspension (ETH) or procaine benzylpenicillin in an oil suspension (UPA); dose in mg/kg; dose frequency (once or twice daily) and treatment duration in days. For example, ETH20-2 × 3 should read as procaine benzylpenicillin in a water suspension 20 mg/kg bodyweight twice daily for three days

The effective treatment time varied between dosing regimens (Fig. 8). A significant association was identified between longer effective treatment time and decreased extent of pneumonia lesions where each hour of additional treatment time resulted in a reduction of 0.30% pneumonia (P < 0.001) and pleuritis by 0.30% (P < 0.001). The percentage of the thorax affected by adhesions was reduced by 0.14% for each additional hour of effective treatment (P = 0.005). No significant association between fever at 48 h post challenge and effective treatment time was observed (P = 0.42).

## Discussion

In the present study, the effect of several different benzylpenicillin dosing regimens for treatment of APP infections in pigs were evaluated and compared with infection in untreated pigs. The dosing regimens that resulted in the highest benzylpenicillin plasma exposure (UPA60 + UPA30-1 × 2, ETH30-2 × 5, ETH30 + UPA30-1 × 3 and UPA30-1 × 5) demonstrated the best clinical efficacy. This was shown not only descriptively for each observed variable, but also by combining the treatment-response ranking of each treatment with the effective treatment time. Despite its wide use in pigs, there is sparse published information about benzylpenicillin’s efficacy against APP in pigs. Therefore, the findings from the present study contribute important information regarding decisions for effective treatment strategies for APP infections and prudent use of narrow-spectrum antibiotics.

This study was designed to provide evidence-based support for the prudent use of antibiotics for effective treatment of APP. The infection model has been previously used by the present research group on several occasions [18, 25]. The model has previously been shown to reproducibly induce clinical signs typical of APP after a predictable time after inoculation [19, 20]. The disease presentations of the untreated animals in the present study were consistent with previous findings which indicate that the bacterial challenge was clinically relevant [19]. Clinical scoring gives an initial indication of the disease status and progression and has been widely used in both clinical practice and under experimental conditions. However, small differences in clinical presentations between treatment groups may not be detectable. There was a variability in effective treatment time for most of the dosing regimens. However, UPA30-1 × 3 displayed greater variability compared with other dosing regimens. The reason behind this variation between individuals in the treatment groups remains unclear. However, sustained release formulations result in slow and erratic absorption, which probably were most pronounced using the UPA30-1 × 3 dosing regimen. Interestingly, this variation was not obvious in the UPA30-1 × 5 treatment group which suggests a large effect of the individual animal.

Benzylpenicillin is considered a time-dependent antibiotic agent [24, 31]. For time-dependent antibiotics, clinical cure has been associated with free plasma concentrations exceeding MIC values for more than 40–50% of the dosing interval [29]. Hence, 50% of the dosing interval (*f*T > 50%) was used as a cut-off value in this study as this is correlated to bactericidal effects which are desired when treating acute diseases such as porcine pleuropneumonia caused by APP. Lower cut-off values have also been correlated with decrease in clinical signs most probably due to a bacteriostatic effect [20, 31].

The present study indicated that there are substantial differences in the overall treatment-response ranking to different dosing regimens. The experimental design allowed re-evaluation of the labelled dose (ETH20-1 × 3) that previously had been associated with inferior treatment efficacy [19, 20]. Moreover, adjusted dosing regimens using higher doses were investigated to evaluate whether an increase in benzylpenicillin exposure were more clinically effective. Despite the use of an established infection model, the present study was experimental and included a limited number of pigs. To reduce the complexity of the statistical analysis, the common measurement of the plasma concentrations of benzylpenicillin was used as an independent variable against lung lesions recorded post-mortem which was considered a cumulative measure of the disease severity in each pig. The benzylpenicillin plasma concentrations were used to calculate an effective treatment time above a therapeutic threshold which was associated with reductions in lesions in the lung. There was also a distinct relationship between treatment regimens with shorter dose intervals or higher individual doses resulting in longer effective treatment time. This statistical method effectively demonstrated that dosing regimes with a long effective treatment time resulted in less severe disease without being able to directly identify the most effective dosing regimen. Given the variation in observed effective treatment time, a plausible difference in 80 h of effective treatment time could result in a 24% decrease in pneumonia and pleurisy lesions and an 11% decrease in thoracic adhesions. Since there were apparent differences in the severity of disease in the two trials and not all treatment regimens were repeated in both trials, the interpretation of the effective treatment time was considered to be more reliable than the more marginal differences between individual treatment regimens which could be confounded by unmeasured inter-trial effects.

Inspection of the overall treatment-response ranking showed that the clinical findings and necropsy results differed between dosing regimens. There was statistical support that increased doses reduced clinical signs, laboratory outcomes, and post-mortem examination findings. The dosing regimens UPA60 + UPA30-1 × 2, ETH30-2 × 5, ETH30 + UPA30-1 × 3 and UPA30-1 × 5 were most effective which indicated that administering a dose above the current labelled dose (10–20 mg/kg once daily) resulted in greater therapeutic success. This was also supported by the effective treatment time as these dosing regimens also led to increased effective plasma exposure for treatment of infections caused by bacteria with a benzylpenicillin MIC of 0.5 mg/L. To evaluate the clinical efficacy of the suggested dosing regimens, clinical field studies are warranted to confirm the results.

Clinical scoring is subjective and needs to be complemented with more objective measures, especially since the clinical scoring was not blinded for practical reasons. In the current study, there were no apparent differences between the different dosing regimens, only between untreated pigs and treated pigs. However, there was an unexpected difference between the observer’s impression of the onset of disease in the two trials where in trial 1, disease onset was perceived as more clinically acute and one pig died peracutely between observations in trial 1. This apparent difference in clinical presentation between the trials could not be supported by statistical differences apart from higher rectal temperatures in trial 1. The rectal temperature, WBC, and SAA are objective measures of inflammatory response and indirectly the burden of infection. In general, all treatment regimens were associated with weaker inflammatory responses compared with untreated pigs. This suggests that all the dosing regimens inhibited bacterial growth. The dosing regimens with long effective treatment time tended to result in a lower inflammatory response compared to those with shorter effective treatment times. This conclusion was also supported by the antibody response where dosing regimens with long effective treatment time resulted in lower antibody response. This suggested that the immune response was lower in pigs that received an effective treatment which likely reduced the pathogen burden. Initiating treatment early in the disease process may result in insufficient immunological response which can lead to a retained susceptibility to APP [19]. However, as all pigs developed antibodies to APP, the risk could be considered low when using the dosing regimens presented in this study. Average daily weight gain was not significantly different between the treatment groups. However, the weight gain was only measured during this grower pig phase and this result of weight gain reduction may not become apparent until later in the finisher period [1].

Even though benzylpenicillin treatment was apparently clinically effective, lung samples and lung abscesses were culture-positive in several pigs from different treatment groups showing that bacterial cure was not achieved. Such pigs could under field conditions serve as subclinical carriers maintaining the infection at herd level with possible future outbreaks of APP. Not even a treatment duration of five days with an increased dose to 30 mg/kg twice daily was sufficient to achieve bacterial cure. However, the extent of lung lesions was less in treated pigs compared to untreated pigs. Therefore, benzylpenicillin may not be the drug of choice to achieve eradication of APP from a herd. Enrofloxacin has been shown to be effective [18, 19, 32] but has also been shown to lead to resistance development in *Escherichia coli* [33].

The results from this study can be extrapolated to infections in other locations or that are caused by different bacteria under certain conditions. The infecting bacteria should be susceptible to benzylpenicillin, and the MIC should not exceed 0.5 mg/L to allow extrapolation from the results of the present study. Moreover, the site of infections should be well-perfused and without any physiological barriers. Benzylpenicillin has a small volume of distribution and does not diffuse into the cell or over *e.g.* the blood–brain barrier [34–36]. Although an inflammation increases the diffusion of benzylpenicillin to the cerebrospinal fluid, the results from the present study cannot be extrapolated to nervous system infections [35]. Therefore, using benzylpenicillin against conditions such as meningitis warrants further investigation of relatively insensitive bacteria (*e.g.* MIC = 0.5 mg/L).

Revised dosing regimens could cause difficulties in the field. During an APP outbreak, administering penicillin twice daily would be practically challenging. To tackle this problem, UPA30-1 × 5 would be feasible. This dosing regimen upheld free plasma concentrations above 0.5 mg/L during most of the dosing intervals [25], which also was confirmed by the calculation of effective treatment time in the present study. Alternate dosing regimens based on doses once daily could be ETH30 + UPA30-1 × 3 or UPA60 + UPA30-1 × 3 which both resulted in acceptable effective treatment time and high overall treatment-response ranking. However, the use of loading doses was considered off-label treatment that requires adjustment of the withdrawal times for slaughter. Hence, those dosing regimens were considered less feasible than UPA30-1 × 5 which is a labelled regimen. Alternatively, other antibiotics can be used to treat APP. Some of these include amoxicillin, tetracycline, fluoroquinolones, and semisynthetic macrolides such as tulathromycin [18, 19, 37–40]. Since these substances have a broader spectrum of antibacterial activity than benzylpenicillin, they also have a larger impact on the intestinal microbiome and hence pose a greater risk for the development of resistance [8, 33, 41–43]. Moreover, those antibiotics categorised as C (macrolides) and B (fluoroquinolones) by EMA-AMEG (European Medicines Agency Antimicrobial Advice Ad hoc Expert Group) should only be used if category D antibiotics (such as benzylpenicillin, amoxicillin, and tetracycline) are not therapeutically effective.

## Conclusions

The results from this study indicated that several dosing regimens, resulting in increased plasma exposure, were associated with improved clinical efficacy than the labelled dose. Although the dosing regimen is partly dependent on the pharmaceutical formulation, which affects the plasma absorption and disposition of benzylpenicillin, a higher dose that increases the time at effective plasma concentrations is motivated. Optimising the use of existing antibiotics is crucial given the limited development of new antimicrobial agents and the need to combat antimicrobial resistance in both human and animal health. The overall treatment-response ranking and the resulting plasma benzylpenicillin exposure showed that penicillin therapy remains a clinically effective treatment strategy for APP.

## Data Availability

The datasets generated and/or analysed during the current study are available in the Zenodo repository, https://doi.org/10.5281/zenodo.14866428.

## Declarations

### Prior publication

Data included in this article have previously been published in the Proceedings of the European Symposium of Porcine Health Management, Prague, The Czech Republic, 3–5 May 2017, and the International Pig Veterinary Society Congress, Chongqing, China June 11–14 2018.
